# Genotype-by-Environment Interactions and Adaptation to Local Temperature Affect Immunity and Fecundity in *Drosophila melanogaster*

**DOI:** 10.1371/journal.ppat.1000025

**Published:** 2008-03-14

**Authors:** Brian P. Lazzaro, Heather A. Flores, James G. Lorigan, Christopher P. Yourth

**Affiliations:** 1 Department of Entomology, Cornell University, Ithaca, New York, United States of America; 2 Field of Genetics and Development, Cornell University, Ithaca, New York, United States of America; Stanford University, United States of America

## Abstract

Natural populations of most organisms harbor substantial genetic variation for resistance to infection. The continued existence of such variation is unexpected under simple evolutionary models that either posit direct and continuous natural selection on the immune system or an evolved life history “balance” between immunity and other fitness traits in a constant environment. However, both local adaptation to heterogeneous environments and genotype-by-environment interactions can maintain genetic variation in a species. In this study, we test *Drosophila melanogaster* genotypes sampled from tropical Africa, temperate northeastern North America, and semi-tropical southeastern North America for resistance to bacterial infection and fecundity at three different environmental temperatures. Environmental temperature had absolute effects on all traits, but there were also marked genotype-by-environment interactions that may limit the global efficiency of natural selection on both traits. African flies performed more poorly than North American flies in both immunity and fecundity at the lowest temperature, but not at the higher temperatures, suggesting that the African population is maladapted to low temperature. In contrast, there was no evidence for clinal variation driven by thermal adaptation within North America for either trait. Resistance to infection and reproductive success were generally uncorrelated across genotypes, so this study finds no evidence for a fitness tradeoff between immunity and fecundity under the conditions tested. Both local adaptation to geographically heterogeneous environments and genotype-by-environment interactions may explain the persistence of genetic variation for resistance to infection in natural populations.

## Author Summary

Genetic variation for resistance to infection is ubiquitous in natural animal and plant populations. This observation runs counter to intuition that resistance should be an important determinant of fitness, and that alleles conferring low resistance should be eliminated by natural selection. We use the model insect *Drosophila melanogaster* to test the hypotheses that species-wide genetic variation for resistance may be maintained by a) adaptation of subpopulations to their local environmental conditions (temperature), b) genotype-by-environment interactions (GxE) determining resistance, and c) correlated fitness costs of resistance, or life history tradeoffs. We measure resistance to bacterial infection and fecundity at three experimental temperatures in *D. melanogaster* collected from three environmentally distinct subpopulations. Indeed, we find that flies from a tropical African subpopulation are, on average, less resistant to infection and less fecund at low temperature than are flies from temperate and semi-temperate populations in North America. We observe considerable GxE for both traits in all populations. Although individual flies are less fecund when they have more severe infections, the genetic correlations between resistance and fecundity are either positive or nonsignificant under our experimental conditions, so we do not find evidence of a life history tradeoff. We conclude that adaptation to local abiotic environment and GxE may maintain species-wide genetic variation for resistance to infection (and fecundity) in *D. melanogaster*, and by logical extension, other species.

## Introduction

Phenotypic traits are determined by a combination of genetic and environmental influences. Even traits that have strong genetic determination can be profoundly influenced by environmental conditions, such that the same genotype may yield quantitatively or qualitatively different phenotypes in different environments. Additionally, distinct genotypes may vary in the degree to which their phenotypes are affected by environmental conditions, termed genotype-by-environment interaction (GxE). In a heterogeneous environment, GxE reduces the population-level correspondence between genotype and phenotype. Since natural selection acts on phenotypes but evolution occurs only through genetic change in populations, GxE reduces the global efficiency of natural selection and can even result in the maintenance of polymorphism.

If environmental heterogeneity is structured across a species range, such that some subpopulations consistently experience different environments than others do, subpopulations may become adapted to their local environmental conditions. Natural selection will favor genotypes that confer the highest fitness in each environment, allowing subpopulations to become genetically and phenotypically differentiated. Local adaptation is particularly efficient when gene flow among subpopulations is low, allowing selection to effectively promote high-fitness alleles in each subpopulation with minimal interference from the immigration of low-fitness alleles from other subpopulations. Local adaptation can also result in maintenance of polymorphism at the species level.

Ambient temperature, like many environmental factors, varies seasonally and geographically. In the fruit fly *Drosophila melanogaster*, latitudinal clines in allele frequencies and phenotypic traits have been attributed to adaptation of equatorial populations to warm climatic temperatures and of temperate populations to cooler and more variable climates. Temperate and tropical *D. melanogaster* subpopulations have been shown to genetically differ for a variety of traits, including body size (*e.g.*, [1]–[3]), diapause [4],[5], fecundity [3] and a suite of metabolic characters (*e.g.*, [6],[7]).

Immune defense against infection is thought to be an important component of fitness in most organisms, and one that can be strongly impacted by environmental conditions. Temperature can have substantial influence on the quality of defense against infection [8]. In ectotherms, environmental temperature can be especially influential on host-pathogen interactions because ambient temperature determines the physiological temperature of both the host and the pathogen [9]–[11], and insects are known to behaviorally manipulate their body temperatures in response to infection (*e.g.*, [12],[13]). Genotype-by-temperature interactions have previously been shown to affect susceptibility to bacterial infection in the crustacean *Daphnia magna* [10].

In this study, we explore the effects of ambient temperature on suppression of bacterial infection in *Drosophila melanogaster*. We specifically a>k whether *D. melanogaster* subpopulations are immunologically adapted to local thermal conditions and whether genotype-by-environment interactions influence relative immune performance. We generated outbred *D. melanogaster* genotypes representing three geographic subpopulations and tested them for the ability to suppress infection by the bacterium *Providencia rettgeri* at experimental temperatures of 18°C, 23°C and 28°C. The sampled *D. melanogaster* subpopulations are resident in New York, northeastern United States, North America (−4°C mean January temperature, 20°C mean July temperature), Georgia, southeastern United States (5°C mean January temperature, 26°C mean July temperature) and Congo, Africa (26°C mean January temperature, 22°C mean July temperature; temperature ranges obtained from http://www.worldclimate.com and http://www.weatherbase.com). We expect environmental temperature to have absolute effects on the host-pathogen interaction, but we are especially interested in whether different populations or genotypes show differential performance under the various temperatures. If the *D. melanogaster* subpopulations are thermally adapted with respect to immunity, subpopulations from warmer climes should have higher resistance to infection at high temperature, and the reciprocal should be true at low temperature.

Immunity has frequently been observed to be costly in *Drosophila* and other insects, and increased immunocompetence is often coupled with decreased performance in other fitness components (*e.g.*, [14]–[18]) although the costs of immunity are frequently observed only in stressful environments (*e.g.*, [16],[19],[20]). Life history tradeoffs between immunity and other fitness determinants can also result in maintained polymorphism if infection pressure is spatially or temporally variable. We therefore measure fecundity in both infected and uninfected flies from each population at all temperatures to assess whether immunity is a costly fitness trait. A negative correlation between resistance and fecundity would support the hypothesis of a life history tradeoff between immunity and other components of fitness.

## Results

Experimental temperature had a profound effect on the outcome of infection. Mortality of *D. melanogaster* infected with *P. rettgeri* was significantly higher at 28°C than at any other temperature (?^2^_(2)_ = 38.09; *p*<0.001). At 28°C, 38.7% of the *P. rettgeri* infected flies died within 48 hours, whereas only 11.4% and 10.9% of the infected flies died at 23°C and 18°C. There was no difference among populations in *D. melanogaster* mortality at 23°C and 28°C, but Congolese flies were significantly more likely to die than North American flies at 18°C (?^2^_(2)_ = 8.97; *p* = 0.011). There was little or no mortality in sham infected or anesthetized flies at any temperature (<5% in each treatment-temperature combinations).

**Figure 1:**
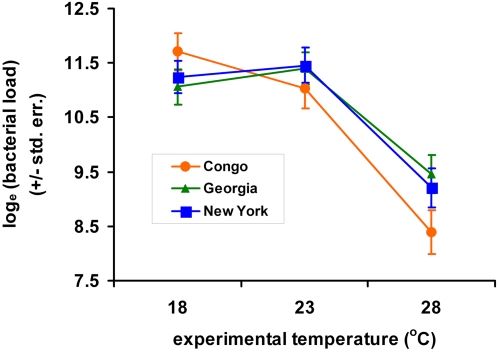
Mean bacterial loads sustained by *D. melanogaster* infected with *P. rettgeri* at three different experimental temperatures. The flies were isolated from natural populations in the Congo, tropical Africa (orange circles), Georgia, southeastern United States (green triangles), and New York, northeastern United States (blue squares). The plotted data are least squares means for each population (Model A, see Materials and Methods) and error bars represent one standard error. The population-by-temperature interaction is significant, *p* = 0.0055.

**Figure 2:**
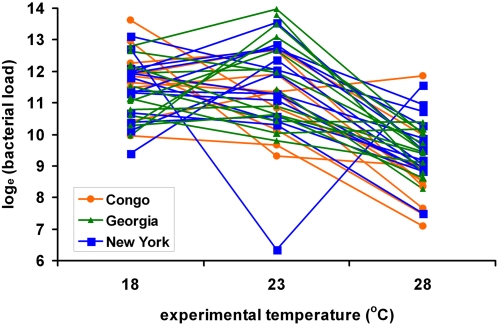
Genotype-by-environment interactions in bacterial load sustained after infection at three experimental temperatures. Least squares mean bacterial loads (Model B, see Materials and Methods) are plotted for *D. melanogaster* genotypes isolated from Congo, Africa (orange circles), Georgia, USA (green triangles), and New York, USA (blue squares). The genotype-by-temperature interaction is highly significant, *p* = 0.0015. Removal of the outlier point at 23°C does not meaningfully change the results.

Surprisingly, bacterial loads sustained by flies from all populations were significantly lower when the flies were reared at 28°C than when the flies were reared at 18°C and 23°C (*p*<0.0001, Models A and B, defined in Materials and Methods; Figures 1 and 2). This observation is unexpected given that *D. melanogaster* post-infection mortality is significantly greater at 28°C than at the other temperatures. Since bacterial loads could not be estimated from flies that died during the experiment (see Materials and Methods), the loads estimated from surviving flies may underestimate the true bacterial loads if the subset of flies that died were those carrying the most bacteria. Mean bacterial load would have been most severely underestimated at 28°C, where 30–40% of the infected flies died, compared to 10–20% mortality at the other temperatures. Even so, we would not expect to see a mean reduction in bacterial load at 28°C unless the true phenotypic distribution was sharply bimodal or unless systemic bacterial loads that are sublethal at 18°C and 23°C become lethal at 28°C. We have no evidence for bimodality in the phenotypic distribution and favor the hypothesis that measurably fewer bacteria are sufficient to kill flies at 28°C, probably because flies reared at higher temperatures are smaller (discussed below).

The African and North American populations responded differently to alteration of the experimental temperature, revealed as a significant population-by-temperature interaction (*p* = 0.006, Model A; Table 1). Congolese flies reared at 18°C sustained higher bacterial loads than flies from either population in North America. In contrast, Congolese flies reared at 23°C and 28°C sustained lower systemic bacterial loads than did North American flies (Figure 1). Even within populations, there was significant heterogeneity among *D. melanogaster* genotypes in *P. rettgeri* load sustained 48 hours post-infection (*p*<0.001, estimated by running Model A but declaring CROSS_g_(POP_p_) to be a fixed effect) and a significant genotype-by-temperature interaction (*p* = 0.002, Model B; Table 2). Our entire experiment was performed in duplicate so we could estimate whether these genotype-by-temperature interactions are repeatable or whether they represent essentially unpredictable noise. The three-way (CROSS*TEMP*DAY)_gtd_ interaction was nonsignificant (*p* = 0.602) in a derivative of Model B that included this factor as a fixed effect. Thus, it appears that the genotype-by-temperature interaction is stable across replicate days and that the phenotype yielded by each genotype is predictable within each environment even if it is variable across environments.

**Table 1:** Results from ANOVA Model A describing the effects of population of origin and rearing temperature on immunocompetence (see Materials and Methods).

Effect	d.f.	F-ratio	*p*-value
Population	2	0.43	0.6528
Temperature	2	71.69	<0.0001
Sex	1	2.02	0.1558
Infector	4	4.21	0.0023
Plater	1	2.67	0.1026
Temp*Pop	4	3.706	0.0055

**Table 2:** Results from ANOVA Model B describing genotype by environment interactions in immunocompetence (see Materials and Methods).

Effect	d.f.	F-ratio	*p*-value
Cross	38	2.32	<0.0001
Temperature	2	51.42	0.0048
Sex	1	1.83	0.1762
Infector	4	3.78	0.0049
Plater	1	2.71	0.1004
Temp*Cross	71	1.64	0.0015

**Figure 3:**
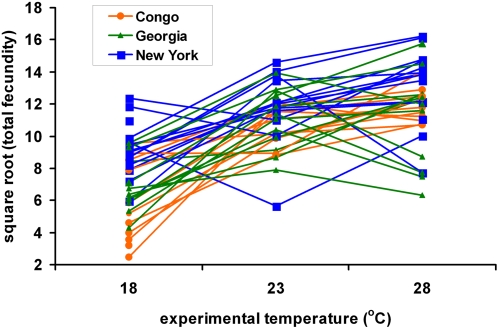
Genotype-by-environment interactions in fecundity of *D. melanogaster* genotypes at three experimental temperatures. Least squares mean fecundities (Model D, see Materials and Methods) are plotted for *D. melanogaster* genotypes isolated from Congo, Africa (orange circles), Georgia, USA (green triangles), and New York, USA (blue squares). Plotted data are the square root of the total fecundity over the 48 hour experimental period for flies that were anesthetized with CO_2_ but otherwise uninjured. The genotype-by-temperature interaction is highly significant, *p*<0.0001.

Fecundity was measured for each cross as the number of progeny that survived to adulthood. Total fecundity was divided into eggs laid in the first 24 hours post-infection and eggs laid in the second 24 hours post-infe>tion. The three populations were highly significantly different in total fecundity (*p*<0.001; Table 3) with flies from New York exhibiting higher fecundity than flies from Georgia or the Congo at all days and temperatures. Flies from Georgia and the Congo had similar fecundities at 23°C and 28°C, but flies from the Congo demonstrated 20% lower fecundity than Georgia flies at 18°C (Table 4). There were also highly significant genotype-by-temperature interactions in both the first and second 24 hour periods following infection (*p*<0.001, Model D; Figure 3). Treatment with the different infection regimes (anesthesia, sterile wound, infection) did not cause any difference in fecundity for the first 24 hours post-infection (*p* = 0.332, Model C; *p* = 0.262, Model D), but fecundities over the second 24 hours post-infection were universally and highly significantly lower in infected flies than in anesthetized or sterilely wounded controls (*p*<0.001, Models C and Model D; Table 4). Interestingly, flies maintained at 18°C laid fewer eggs in the first 24 hours post-infection than they did in the second 24 hours, but flies reared at 28°C showed the opposite pattern (Table 4).

**Table 3:** Results from ANOVA Model C describing fecundity at 18°C, 23°C, and 28°C (see Materials and Methods).

		First 24 hours		Second 24 hours		Total	
Population	d.f	F-ratio	*p*-value	F-ratio	*p*-value	F-ratio	*p*-value
Population	2	9.52	0.0005	19.14	<0.0001	14.35	<0.0001
Temperature	2	406.17	0.0002	0>95	<0.4788	21.50	0.0167
Treatment	2	1.11	0.3317	10.66	<0.0001	3.66	0.0263
Infector	3	0.10	0.9575	13.10	<0.0001	3.79	0.0102
Females	1	37.49	<0.0001	113.96	<0.0001	74.41	<0.0001
Pop*Temp	4	3.75	0.0050	4.42	0.0016	3.74	0.0051
Least squares mean fecundities, square root transformed (std. err.)							
Congo		6.23 (0.356)		5.93 (0.457)		8.84 (0.455)	
Georgia		6.21 (0.308)		6.27 (0.447)		9.04 (0.417)	
New York		7.84 (0.293)		7.50 (0.442)		11.06 (0.404)	

**Table 4:** Untransformed mean (std. dev.) fecundity of *D. melanogaster* maintained at 18°C, 23°C, and 28°C and subjected to different infection regimes.

Population	Treatment	Time Period	18°C	23°C	28°C
Congo	CO_2_ anesthesia	first 24 hours	12.85 (13.11)	51.38 (22.11)	86.37 (30.16)
		second 24 hours	32.00 (29.84)	54.67 (29.41)	59.35 (21.55)
	sterile wound	first 24 hours	15.80 (14.03)	55.25 (25.62)	89.95 (26.78)
		second 24 hours	31.79 (29.91)	54.94 (32.51)	60.35 (16.75)
	*P. rettgeri* infection	first 24 hours	11.55 (10.90)	50.71 (25.05)	68.28 (27.47)
		second 24 hours	24.68 (22.39)	38.73 (27.88)	34.92 (23.89)*
Georgia	CO_2_ anesthesia	first 24 hours	17.09 (16.84)	58.00 (35.69)	85.67 (45.94)
		second 24 hours	37.10 (25.37)	56.71 (38.55)	59.83 (26.28)
	sterile wound	first 24 hours	20.59 (21.50)	53.14 (30.03)	73.65 (44.44)
		second 24 hours	44.00 (27.87)	50.09 (20.58)	58.91 (28.29)
	*P. rettgeri* infection	first 24 hours	20.61 (21.69)	49.19 (25.39)	71.77 (40.63)
		second 24 hours	38.00 (25.50)	38.71 (23.86)	38.09 (21.72)*
New York	CO_2_ anesthesia	first 24 hours	33.10 (24.43)	75.08 (35.48)	109.59 (39.64)
		second 24 hours	60.71 (33.58)	69.52 (37.37)	79.29 (32.21)
	sterile wound	first 24 hours	36.55 (21.16)	78.21 (35.62)	108.92 (41.52)
		second 24 hours	70.71 (35.32)	70.25 (37.76)	73.89 (32.85)
	*P. rettgeri* infection	first 24 hours	29.75 (18.75)	79.46 (29.81)	87.43 (43.82)
		second 24 hours	51.89 (29.25)	58.95 (25.12)	47.33 (32.23)*

*: Fecundities reported in this table for the second 24 hour period following infection with *P. rettgeri* at 28°C may be slight underestimates because they are uncorrected for female mortality.

All populations harbored highly significant genetic variation for both immunocompetence and fecundity, prompting a test of whether variation in these traits was genetically correlated. A positive genetic correlation between fecundity and bacterial load sustained after infection (*i.e.*, a negative correlation between fecundity and resistance) would indicate a fitness cost of increased immunity. Alternatively, a negative genetic correlation between fecundity and bacterial load sustained (a positive correlation between fecundity and resistance) would indicate differences in overall fitness among genotypes. In these analyses, it is of interest to measure the relationship between bacterial load sustained and fecundity of both infected and uninfected flies. The genetic correlation between fecundity of uninfected flies and resistance to infection provides a measure of the constitutive cost of i>munity. The correlation between immunity and fecundity of infected flies describes the ability of different genotypes to produce offspring while managing an active infection. Because both sexes are infected in our experimental design, the effects of infection on male and female reproductive traits are confounded in the global estimate of fecundity.

**Figure 4:**
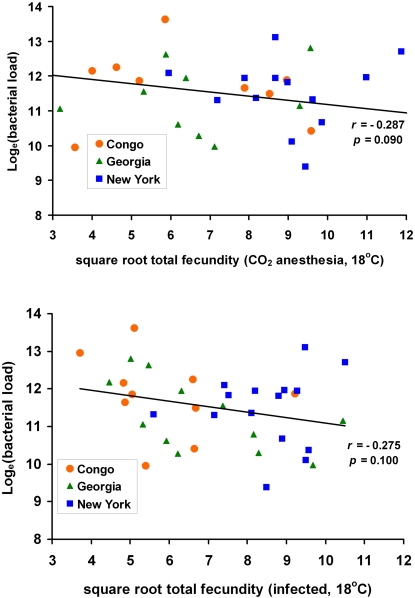
Genetic correlations between immunity and fecundity at the 18°C experimental temperature. For each genotype, total fecundity of CO_2_ anesthetized flies (upper panel) or total fecundity of infected flies (lower panel) is plotted against bacterial load sustained by infected flies of that genotype. Thus, bacterial load and fecundity of infected flies are measured on the same individuals. Fecundity of CO_2_ anesthetized flies is compared to load sustained by distinct infected flies of the same genotype. The best fit regression line for the data including all three populations is plotted. The correlation is negative in both cases, indicating that some genotypes are generally unfit, experiencing both high pathogen loads and low fecundities. Genotypes from the Congo (orange circles) perform particularly poorly at 18°C. There is no correlation between immunity and fecundity at 23°C or 28°C. There is no evidence for a life history tradeoff between immunity and fecundity, which would be revealed as a positive relationship between bacterial load sustained and total fecundity.

The severity of infection in individual *D. melanogaster* females had a significant effect on fecundity 24–48 hours after infection at all three temperatures and without respect to genotype (*F*_(1)_ = 6.03, *p* = 0.015). Higher infection intensities resulted in lower fecundity, suggesting that fecundity of individual flies suffers as a consequence of their infection and demonstrating a physiological cost of infection. It is necessary to examine the correlation across genotypes, however, to detect genetic tradeoffs. Looking at genetic correlations, we find a negative trend relating mean bacterial load sustained and mean total fecundity in CO_2_ anesthetized flies (*r* = −0.287, *p* = 0.090) at a rearing and infection temperature of 18°C. The same trend appears when flies are infected with *P. rettgeri* at 18°C (*r* = −0.275, *p* = 0.100). In the second 24 hour period following infection, where infection status had a strong absolute effect on fecundity, this correlation becomes marginally significant (*r* = −0.324, *p* = 0.050). The negative genetic correlations indicate that some genotypes both have low fecundity and suffer high bacterial loads, and hence are generally unfit, at 18°C. These genotypes are disproportionately derived from the Congo population (Figure 4). Bacterial load sustained and fecundity are completely uncorrelated genetically at rearing and infection temperatures of 23°C and 28°C. In no case did we find evidence of a fitness tradeoff between immunity and fecundity.

## Discussion

Natural populations frequently exhibit genetic variation for important fitness traits like immunity, which may seem counter to the expectation that natural selection should favor alleles that maximize fitness. Genetic variation can be maintained, however, if environmental conditions vary across a species range and if distinct alleles confer the highest fitness under each environment. Ge>otype-by-environment interactions erode the efficiency of natural selection, and may even maintain polymorphism, by partially decoupling genotype and phenotype over all environments. Previous work has shown that natural *D. melanogaster* populations harbor substantial genetic variation for the ability to combat microbial infection [20]–[23]. In this study, we evaluated the effects of ambient temperature on *D. melanogaster* immune performance in order to determine whether thermal adaptation in *D. melanogaster* subpopulations and genotype-by-environment interactions may contribute to species-wide genetic variability in immunocompetence. Genetic and phenotypic correlations between immunity and fecundity were also measured at all temperatures in order to determine whether variation in immunity may be maintained by fitness tradeoffs.

As expected, environmental temperature had a strong effect on immunity phenotypes. Flies reared and infected with *P. rettgeri* at 28°C were much more likely to die from the infection than were genetically identical flies reared and infected at 23°C or 18°C. Elevated temperature decreases time to death in *D. melanogaster* infected with other bacterial pathogens [11], so this result is unsurprising. Unexpectedly, however, flies reared at 28°C sustained much lower bacterial loads than did flies reared at 18°C or 23°C. The mechanistic basis for the disconnection between bacterial proliferation and host mortality at different temperatures is unknown. Overstimulation of the immune system in response to infection can hasten death in *Drosophila* [24], and it is possible that the *D. melanogaster* immune system is more active at 28°C, better controlling bacterial proliferation but at the expense of increased host mortality. *D. melanogaster* reared at high temperature develop more quickly and are smaller than genetically identical flies reared at lower temperatures [1]–[3], so a perhaps more likely explanation is that smaller flies sustain fewer bacteria but are also less able to tolerate infection. We did not measure body size in this experiment so we cannot directly test this hypothesis. If true, however, this would indicate an effect of environment not just on the host immune system but on the host's ability to withstand infection. *P. rettgeri* grows well in culture at temperatures at least as high as 37°C (unpublished observation) so we assume that the bacteria suffer no growth defects at 28°C in the fly. We cannot exclude the possibility that the bacteria produce a lethal toxin at 28°C that is not produced at lower temperatures, but neither do we have any evidence supporting this hypothesis. Environmental conditions most probably affect host-pathogen interactions through a complex mediation of host immunity, host tolerance and pathogen virulence. Regardless of mechanism, there is a clear effect of environmental temperature on the course of bacterial infection in *D. melanogaster*, and this effect is likely to be important in natural settings.

Variation in temperature also affected *D. melanogaster* fecundity, irrespective of immune status. Total fecundity increased with increasing temperature, though the distribution of eggs laid over the two measured days was not equivalent across temperatures. When flies were reared at 18°C, they produced many fewer progeny in the first 24 hours of the assay period than they did in the second 24 hours of observation. When flies we>e reared at 23°C, fecundity was approximately equal between the two days. But when flies were reared at 28°C, fecundity was far greater in the first 24 hours than it was over the second 24 hour period. This pattern was observed in infected, sterilely wounded, and CO_2_ anesthetized flies. Nevertheless, fecundity was impacted by infection, and the fecundities of individual females are significantly increasingly reduced with increasing severity of infection. *D. melanogaster* infected with *P. rettgeri* produced significantly fewer progeny than their uninfected counterparts, especially in the second 24 hour period post-challenge. Similar results have previously been observed by Brandt and Schneider [25], who demonstrated that some bacteria limit egg production by colonizing *Drosophila* ovaries and resulting in their degeneration. Zerofsky *et al.* [26] have suggested that activation of an immune response alone causes a reduction in egg production, and that the effect persists through the remainder of life. We terminated our experiment after two days of egg laying, so the effects of environmental temperature and infection on lifetime fecundity of the fly are unknown. It is clear, however, that infection causes a marked decrease in reproductive potential, at least over the short term.

All three populations carry substantial genetic variation for both immunity and fecundity, and there were significant genotype-by-environment interactions in both traits. Nevertheless, phenotypic differentiation between African and North American flies for both traits suggests thermal adaptation in these subpopulations. In particular, flies from the Congo were the best able to suppress *P. rettgeri* proliferation at the highest temperature, 28°C. On the other hand, flies from the Congo sustained higher bacterial loads than did flies from North America at 18°C (Figure 1). Congolese flies also exhibited significantly lower fecundity at 18°C than did North American flies, consistent with a previous report that tropical *D. melanogaster* are less fit than temperate flies at low temperature [3]. *D. melanogaster* living in the Congo are not subject to the seasonally low environmental temperatures that North American *D. melanogaster* are. It is likely that the Congo population lacks genetic variants that perform well at lower temperatures, but that such variation is selectively maintained in the North American populations. Replicate sampling of thermally equivalent populations will be required to thoroughly test this hypothesis. African and non-African *D. melanogaster* have previously been shown to differ in resistance to fungal infection, with African flies suffering higher mortality after exposure to fungal spores at 25°C [23]. Genetic differentiation in immunocompetence between African and non-African flies may be more common than is generally appreciated. The flies derived from New York and Georgia populations are very similar to each other in immunity phenotypes at all three temperatures examined here. This suggests that, as far as immunity is concerned, these two populations may be considered undifferentiated and does not support a hypothesis of clinal variation in immunity in eastern North America.

Unexpectedly, the flies from New York exhibited higher fecundity than did the flies from Georgia and the Congo at all temperatures and under all infection treatments. Georgia and Congo flies were indistinguishable in fecundity at 23°C and 28°C, although as mentioned above, Congo flies suffer reduced fecundity at 18°C. *D. melanogas>er* are known to vary clinally and geographically for body size [1]–[3],[27],[28] and increased body size is often correlated with increased egg production. Although body size is phenotypically plastic, *D. melanogaster* derived from colder locales tend to remain larger than their temperate counterparts when reared under a range of environmental conditions [28],[29]. Thus, it is possible that the New York flies are more fecund than the other flies in the study because they are genetically larger. If this is the case, however, the body size effect does not extend to immunity phenotypes. A perhaps more likely explanation may be that the New York flies are pre-adapted to the experimental conditions. Each isofemale line was created and maintained by mass sib-mating the progeny from a single wild female, potentially allowing adaptation to laboratory conditions during the first generations of inbreeding. The New York isofemale lines were established on the medium used in this study, whereas the isofemale lines from the other populations were inbred on different media in different laboratories. Thus, the hyper-fecundity of the New York flies may be explained if the New York flies are better adapted to the *Drosophila* medium used in this study. If the New York flies are better adapted to the *Drosophila* medium used here, it is only apparent with fecundity measures, as again the New York flies did not generally perform better in immunity phenotypes.

Adaptation to the lab could also result in flies becoming thermally adapted to laboratory temperatures (typically 22°C–25°C). If this had occurred, performance in all phenotypes should be better at 23°C than at 18°C or 28°C. That pattern was not observed, suggesting that adaptation to the lab thermal environment did not impact our experiment. In any case, adaptation to an intermediate temperature would be conservative with respect to discovering genetic variation in the sampled flies and would cause our experiment to underestimate genetic differentiation within and between populations.

Previous studies have documented negative genetic and phenotypic correlations between reproductive traits and resistance to bacterial infection in *D. melanogaster* [16],[19],[20]. Although infection had a massive effect on the fecundity of infected flies, we saw no genetic correlation between resistance to infection and fecundity aside from the fact that flies from the Congo performed poorly in both traits at the lowest temperature, 18°C. This study thus finds no evidence for a fitness cost of genetically increased immune capacity. The failure to detect a tradeoff differs from the study of McKean *et al.* [20], in which resistance to infection by *P. rettgeri* at 22–24°C was found to be negatively correlated with pre-infection fecundity under conditions similar to those utilized here. The present study is smaller in scale than the study by McKean *et al.* and so may have less power to detect correlation between the phenotypes. The negative correlation detected in the McKean *et al.* study was eliminated when the nutritional status of the flies was improved by supplementing the diet with yeast, and the inconsistency of the genetic correlations between the two studie> may reflect microenvironmental differences between the experiments. If true, this would underscore the importance of environment in determining phenotypes relevant to fitness [30]. In the present experimental framework, local adaptation and genotype-by-environment interactions appear to be more important than life history tradeoffs for maintaining genetic variation in antibacterial immunity.

We have demonstrated that interactions between *D. melanogaster* genotype and abiotic environment may substantially affect the evolution of resistance to infection. We emphasize, however, that our entire study was conducted using a single genotype of only one pathogen, whereas wild *Drosophila* are naturally exposed to a wide range of genetically diverse pathogens. Host genotype by pathogen genotype interactions (G_H_xG_P_) and even three way interactions between host genotype, pathogen genotype, and the environment (G_H_xG_P_xE) are also important in determining the outcome of infection (*e.g.*, [10]). These effects are particularly important insofar as pathogens may co-evolve with hosts, such that the array of pathogen genotypes may be in continuous flux. The evolution of host resistance in natural populations is likely to be determined by a complex of factors, including pathogen diversity, variability in abiotic environment, and host genetic variation.

Despite the potential for noise in the natural system, we have demonstrated that *D. melanogaster* subpopulations exhibit adaptation to local thermal conditions, which indicates that selection can be effective. The significant genotype-by-environment interactions (GxE) observed with immunity and fecundity phenotypes, however, mean that the rank performance of different genotypes changes across thermal environments. As environmental temperatures vary seasonally and geographically within the *D. melanogaster* species range, GxE probably hinders natural selection and may even promote the selective maintenance of polymorphism. Our data add further evidence that environmental conditions can strongly impact trait expression, and we emphasize that the effects of environmental variation should not be neglected in studies of phenotypic performance and adaptation.

## Materials and Methods

### *Drosophila* and bacterial stocks

Ten *Drosophila melanogaster* isofemale lines established in 2001 from a population in Pointe Noire, Republic of Congo, Africa, (4.5°S, 11.5°E) were obtained from Z. Bochdanovits and A. G. Clark [2]. Fifteen North American isofemale lines established from a population near Athens, Georgia, USA (33.6°N, 83.2°W) in 2003 were obtained from V. Corby-Harris and D. Promislow. Fifteen North American isofemale lines representing a population from Newfield, New York, USA, (42.3°N, 76.3°W), were established by B. P. Lazzaro and E. M. Hill in 2004. Each of the North American isofemale lines was originally generated by mass sib-mating the progeny of a single inseminated female captured in the wild. The Congolese isofemale lines were isolated as progeny of single females pulled from a large laboratory population established from flies collected in the wild [31]. All of the isofemale lines have since been maintained by recurrent mass sib-mating, so they have gradually lost heterozygosity through inbreeding and genetic drift. Flies were maintained at laboratory temperatures (typically 22°C–24°C) during this inbreeding process, so alleles adaptive to extreme temperatures may have been lost during the establishment of the lines. Such loss, if it occu>red, would be conservative with respect to our experiment.

In order to avoid potential phenotypic effects of inbreeding in the isofemale lines, we generated flies to be phenotyped for this experiment using a within-population chain cross design. Within each population, females from line 1 were crossed to males of line 2, females of line 2 were crossed to males of line 3, and so on, establishing 10, 15 and 15 unique crosses for the Congo, New York and Georgia populations. Immunity and fecundity phenotypes were collected from the F1 progeny of each of these crosses. Thus, all phenotyped flies in this experiment are expected to be as heterozygous as any random outbred fly from their respective subpopulations. This crossing design maximizes outbreeding, but may result in slight underestimation of within-population phenotypic variability since the phenotyped F1 progeny from each cross share 50% genetic identity with the F1 progeny from two other crosses in that population set.

The bacterium used for infectious challenge was an isolate of *Providencia rettgeri*. This isolate was collected from the hemolymph of a wild-caught *D. melanogaster* captured in Pennsylvania, USA, approximately 250 kilometers from the location where the New York flies were sampled [32]. We chose this bacterium as an arbitrary natural pathogen of *D. melanogaster* that is amenable to experimental lab work.

### Infection and temperature regime

Resistance to *P. rettgeri* infection and fecundity were measured in the F1 progeny from each of the 40 within-population crosses at 18°C, 23°C, and 28°C. Fecundity was measured after three “infection” treatments: true infection with *P. rettgeri*, sham infection with a sterile needle, and CO_2_ anesthesia without injury. Flies were reared at their experimental temperature from the egg stage and were maintained at that temperature for the duration of the experiment. All flies were maintained throughout the experiment on standard *Drosophila* medium (8.3% w/v glucose, 8.3% w/v brewer's yeast, 1% w/v agar).

Infections, sterile wounds, and anesthesia treatments were applied to groups of three virgin males and three virgin females aged 2–4 days post-eclosion. The six flies in each treatment group were then pooled in a single vial where they mated and laid eggs. *D. melanogaster* mortality was recorded 24 hours after infection, and surviving flies were transferred to vials containing fresh medium. Mortality was recorded again at 48 hours post-infection and surviving flies were removed from the vials for measurement of their immune phenotypes. The 24- and 48-hour vials were retained at their respective temperatures until the next generation of adults emerged. These F2 adults were counted to estimate the fecundity of each F1 cross.

For bacterial infections, *P. rettgeri* was grown overnight at 37°C in LB broth, then diluted to an optical density of A_600_ = 1.0 (±0.1). Infections were delivered by dipping a 0.1 mm minuten pin in the diluted bacteria, then using that pin to pierce the thorax of flies lightly anesthetized with CO_2_. This procedure delivers an average of 5×10^3^ bacteria to each fly (data not shown). Sterile wounds were delivered by pricking the flies with a sterile needle. Anesthetized controls were held on CO_2_ for an equivalent length of time without injury. *D. melanogaster* genotypes were assigned randomly to infectors for treatment on each experimental day.

Bacterial load was estimated from infected *D. melanogaster* that survived 48 hours. Bacterial load was never measured from dead flies since bacteria rapidly proliferate soon after the death of the fly (B.P.>., unpublished observations). Same-sex pairs of flies were homogenized in 500 µl LB broth and homogenates were quantitatively plated on standard LB plates using robotic spiral platers (manufactured by Don Whitley Scientific and Spiral Biotech). Plates were incubated overnight at room temperature. The number of colonies that grew on each plate provided an estimate of mean systemic bacterial load sustained by each pair of flies. This method may yield an underestimate if bacteria exist in the fly as aggregates that are not dispersed by homogenization. We have no reason, however, to think this potential underestimation would in any way bias our experiment. The colonies on 76 of the plates grew too densely for the counter to discriminate. These were coded to take the value of the third-largest measurable data point, an underestimate of their true values. The results are essentially unchanged if these data points are completely eliminated from the analysis (not shown). We consider genotypes that sustained comparatively low systemic bacterial loads to be more “resistant” to infection.

The entire experiment was performed in duplicate, such that each treatment was performed on each genotype on two separate days. Flies from the 18°C and 23°C environments were treated on the same pair of days. Because they developed more quickly, flies reared at 28°C were treated on a distinct pair of days. Bacterial infections were delivered to duplicate groups of 3 males and 3 females from each of the 40 crosses on each day of the experiment. Sham infections and CO_2_ anesthesia treatments were performed on single groups of 3 males and 3 females on each day. Thus, in design, a total of 12 male and 12 female flies from each of the 40 crosses were to be given bacterial infections and 6 flies of each sex were given each control treatment at each of the three temperatures. On one of the replicate days, twelve crosses from the 23°C treatment were accidentally placed at 18°C after being transferred to new medium. These mishandled flies were eliminated from the experiment. An additional small number of data points were dropped because of clerical errors during the experiment or low productivity of the initial cross to establish the F1 test flies. The mishandled flies and lost data were distributed evenly over the three populations.

### Statistical analysis

Mixed model analyses of variance (mixed ANOVA) were performed to detect the effects of *D. melanogaster* genotype, population of origin, experimental temperature, and infection treatment on variance in systemic bacterial load sustained after infection with *P. rettgeri* and fecundity over the experimental period. Statistical interactions between experimental temperature and *D. melanogaster* genotype and population of origin were measured as tests for genotype-by-environment interaction and local adaptation. The effects of other factors, such as the identity of the person performing the infection and the date of infection, were controlled in the analysis. All mixed ANOVA were performed using PROC MIXED in SAS Stat v. 9.1 (SAS Institute, Cary, NC), and the various factors tested in all models are given in Table 5.

**Table 5:** Factors Tested in Analyses of Variance (see Materials and Methods for full models).

Factor	States	Effect Type	Effect Measured	Relevant Models
POP_p_	3	fixed	population from which flies are derived	A, C
TEMP_t_	3	fixed	temperature at which flies were reared and phenotyped	A, B, C, D
SEX_s_	2	fixed	sex of phenotyped flies	A, B
INFECTOR_l_	4	fixed	individual investigator that performed the infections	A, B, C, D
PLATER_k_	2	fixed	which of two instruments was used to plate fly homogenates	A, B
CROSS_g_(POP_p_)	10–15	random	genotypes sampled from each population	A, C
DAY_d_(TEMP_t_)	2	random	replicate day on which experiment was performed	A, B, C, D
CROSS_g_	40	fixed	genotypes of flies phenotyped, irrespective of population	B, D
TREAT_i_	3	fixed	CO_2_ anesthesia, sterile wound, or infection with *P. rettgeri*	C, D
FEMALES_f_	continuous		number of live females in vial at time of fecundity measure	C, D
(POP*TEMP)_pt_	9	fixed	differential effects of temperature on flies from the three populations	A, C
(CROSS*TEMP)_gt_	120	fixed	differential effects of temperature of flies of each genotype	B, D
ε			residual error in each model	A, B, C, D

Four main models were tested. The first model, referred to as Model A in the body of the text, is intended to test population level differentiation in response to infection. Model A takes the form
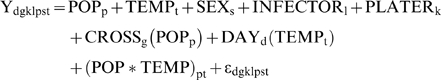
where the response variable, Y, is the natural log of the bacterial load sustained. A similar model, referred to as Model B, was used to test the effect of *D. melanogaster* genotype irrespective of population identity and to test for a genotype-by-temperature interaction. Model B takes the form
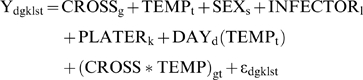
where again the response variable is the natural log of the bacterial load sustained.

Fecundity was measured in every treatment for each cross as the number of F2 progeny to reach adulthood. Fecundity was measured separately for the first and second 24 hour periods following infection, and total fecundity was obtained by summing the fecundities for these two periods. Since absolute fecundity measures were non-normally distributed, these were square-root transformed prior to analysis of vari>nce. Fecundity data were analyzed using two mixed models analogous to Models A and B above. The first model, which will be referred to as Model C, tests whether *D. melanogaster* populations differ in fecundity. This model takes the form
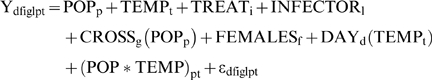
where the Y is the square root of the observed fecundity. An analogous model, which included the bacterial load estimated from each group of females, was used to determine whether severity of infection affected fecundity in individual flies. Effects of genotype irrespective of population and the genotype-by-temperature interaction were tested with Model D, which takes the form
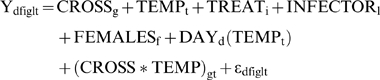
where again Y is the square root of the observed fecundity.

Least squares mean bacterial loads were generated for each cross using Model B, and least squares mean fecundity were generated for each cross using Model D. The relationship between immunity and fecundity across genotypes was evaluated as the correlation between the least squares mean fecundity and least squares mean immunity, determined using PROC REG (SAS Stat v. 9.1). Logistic regression was used to determine whether population of origin and experimental temperature had significantly affected *D. melanogaster* mortality after infection with *P. rettgeri* using PROC LOGISTIC (SAS Stat v. 9.1).
